# A Mobile App for Advance Care Planning and Advance Directives (Accordons-nous): Development and Usability Study

**DOI:** 10.2196/34626

**Published:** 2022-04-20

**Authors:** Céline Schöpfer, Frederic Ehrler, Antoine Berger, Catherine Bollondi Pauly, Laurence Buytaert, Camille De La Serna, Florence Hartheiser, Thomas Fassier, Christine Clavien

**Affiliations:** 1 Institute for Ethics, History, and the Humanities University Medical Center University of Geneva Geneva Switzerland; 2 Department of Medical Information Sciences University Hospitals of Geneva Geneva Switzerland; 3 Direction of Care University Hospitals of Geneva Geneva Switzerland; 4 University Medical Center University of Geneva Geneva Switzerland; 5 Division of Internal Medicine for the Aged University Hospitals of Geneva Geneva Switzerland; 6 Interprofessional Simulation Center Faculty of Medicine University of Geneva Geneva Switzerland

**Keywords:** usability, mobile apps, advance directives, advance care planning, mHealth, mobile health, palliative care, mobile phone

## Abstract

**Background:**

Advance care planning, including advance directives, is an important tool that allows patients to express their preferences for care if they are no longer able to express themselves. We developed *Accordons-nous*, a smartphone app that informs patients about advance care planning and advance directives, facilitates communication on these sensitive topics, and helps patients express their values and preferences for care.

**Objective:**

The first objective of this study is to conduct a usability test of this app. The second objective is to collect users’ critical opinions on the usability and relevance of the tool.

**Methods:**

We conducted a usability test by means of a think-aloud method, asking 10 representative patients to complete 7 browsing tasks. We double coded the filmed sessions to obtain descriptive data on task completion (with or without help), time spent, number of clicks, and the types of problems encountered. We assessed the severity of the problems encountered and identified the modifications needed to address these problems. We evaluated the readability of the app using Scolarius, a French equivalent of the Flesch Reading Ease test. By means of a posttest questionnaire, we asked participants to assess the app’s usability (System Usability Scale), relevance (Mobile App Rating Scale, section F), and whether they would recommend the app to the target groups: patients, health professionals, and patients’ caring relatives.

**Results:**

Participants completed the 7 think-aloud tasks in 80% (56/70) of the cases without any help from the experimenter, in 16% (11/70) of the cases with some help, and failed in 4% (3/70) of the cases. The analysis of failures and difficulties encountered revealed a series of major usability problems that could be addressed with minor modifications to the app. *Accordons-nous* obtained high scores on readability (overall score of 87.4 on Scolarius test, corresponding to elementary school level), usability (85.3/100 on System Usability Scale test), relevance (4.3/5 on the Mobile App Rating Scale, section F), and overall subjective endorsement on 3 *I would recommend* questions (4.7/5).

**Conclusions:**

This usability test helped us make the final changes to our app before its official launch.

## Introduction

### Background

Medical progress increases the availability of treatment options and life-sustaining possibilities. Consequently, it also increases the necessity to make choices between medical options that have different impacts on patients’ future lives. When patients are not capable of making those decisions, health care professionals and surrogates are requested to choose, even if they have no clue about the patient’s values and priorities. To avoid such distressful and suboptimal situations, early advance care planning (ACP) is increasingly being recognized as an important condition for adequate treatment [1,2]. ACP is “the ability to enable individuals to define goals and preferences for future medical treatment and care, to discuss these goals and preferences with family and health care providers, and to record and review these preferences if appropriate” [3]. An ACP procedure may result in written advance directives (AD); however, for such document to be of any use, it remains important that it is precise and that patients discuss its content with their surrogate decision-maker and update it regularly [4].

Despite the acknowledged importance of ACP, few patients discuss their fears, priorities, and the type of care they would like to receive with their families and professional caregivers [1,5,6]. This is partly because of a lack of knowledge about ACP, a lack of recognition that ACP is relevant for them, and a lack of assistance in this psychologically heavy process [6-8]. Studies also indicate that even health care professionals find it difficult to initiate such discussions [9-11].

ACP can be seen as a process of behavior change involving steps, including awareness and knowledge acquisition, thinking and commitment to act (eg, talking to someone and writing AD), action, and regular updates [12,13]. It has been suggested that patient-centered, computer-based infographics could enhance interest, understanding, recalling, contemplation, and actual sharing of decisions related to ACP [14]. They may assist in the ACP process and save clinicians’ time, as patients may be able to obtain relevant information and input to start the ACP process in a family context. Free web-based ACP tools have been developed, with promising preliminary results [15-20]. Mobile apps for ACP are available in some countries. Their potential is recognized; however, existing products have limited features and operate mostly in English only [21,22]. We did not find comparable tools available in French. A recent review concluded that overall, mobile apps provide insufficient content and features: they are helpful to users who are ready to complete AD rather than those seeking to learn about the ACP process. Moreover, they are poor in terms of design quality, layout, and functionality. Only one app (in English) was assessed as “fairly easy to read” [21].

### Accordons-nous

To support the ACP process for French-speaking Swiss residents, our interprofessional research team comprising ethicists, physicians, nurses, patients as partners, information technology professionals developed a free and easy-to-use solution, an app called *Accordons-nous* (*Let’s agree*).

We developed the content and structure of the app between January 2019 and July 2021 using mixed methods—a Delphi procedure and multiple user tests involving laypeople and patients as partners. Multimedia Appendix 1 [23-25] provides the details about the process of making the app.

*Accordons-nous* aims to address known barriers to ACP: lack of awareness; insufficient health literacy; difficulty in starting the discussion; identifying one’s own preferences, values, and goals of care; and writing personalized and comprehensive AD and updating them regularly within a smooth process.

The app is adapted to the local context of French-speaking Switzerland, designed to be used by patients and their family caregivers independently at home or to support ACP discussions with health professionals. Its content is adapted to the health literacy of ordinary patients. It includes (1) content to make users aware of the importance of anticipating health-related decisions and motivate them to engage in their ACP; (2) essential information on the ACP procedure, legal issues, definitions of technical terms in a format, and vocabulary accessible to common users; (3) discussion prompts to help users engage in ACP discussions with their family, friends, and professional care providers; (4) a card game to help patients clarify their values and priorities in life and reflect on the conditions that would make life not worth living; and (5) a detailed and comprehensive advance directive form that can be tailored to individual life and health situations.

**Figure 1:**
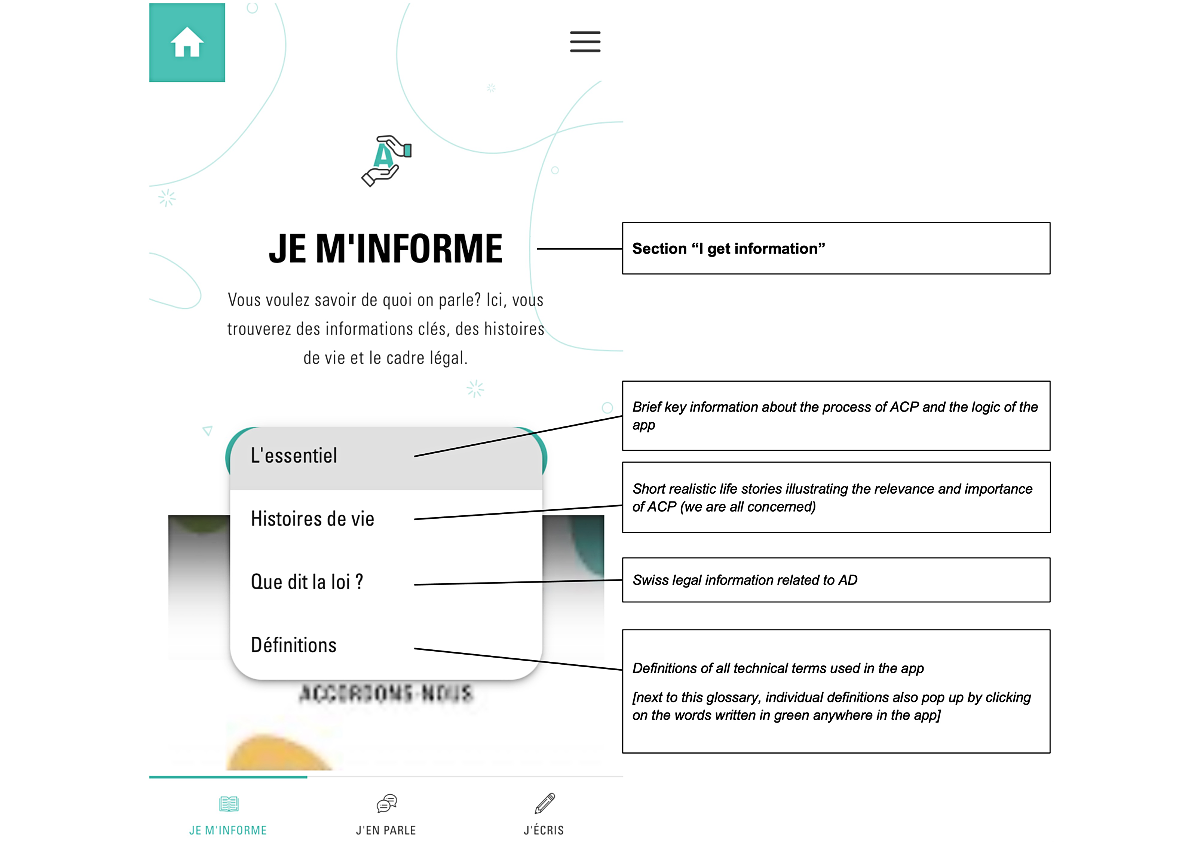
Structure of the section I get information containing subpages accessible through a drop-down menu. ACP: advance care planning; AD: advance directives.

**Figure 2:**
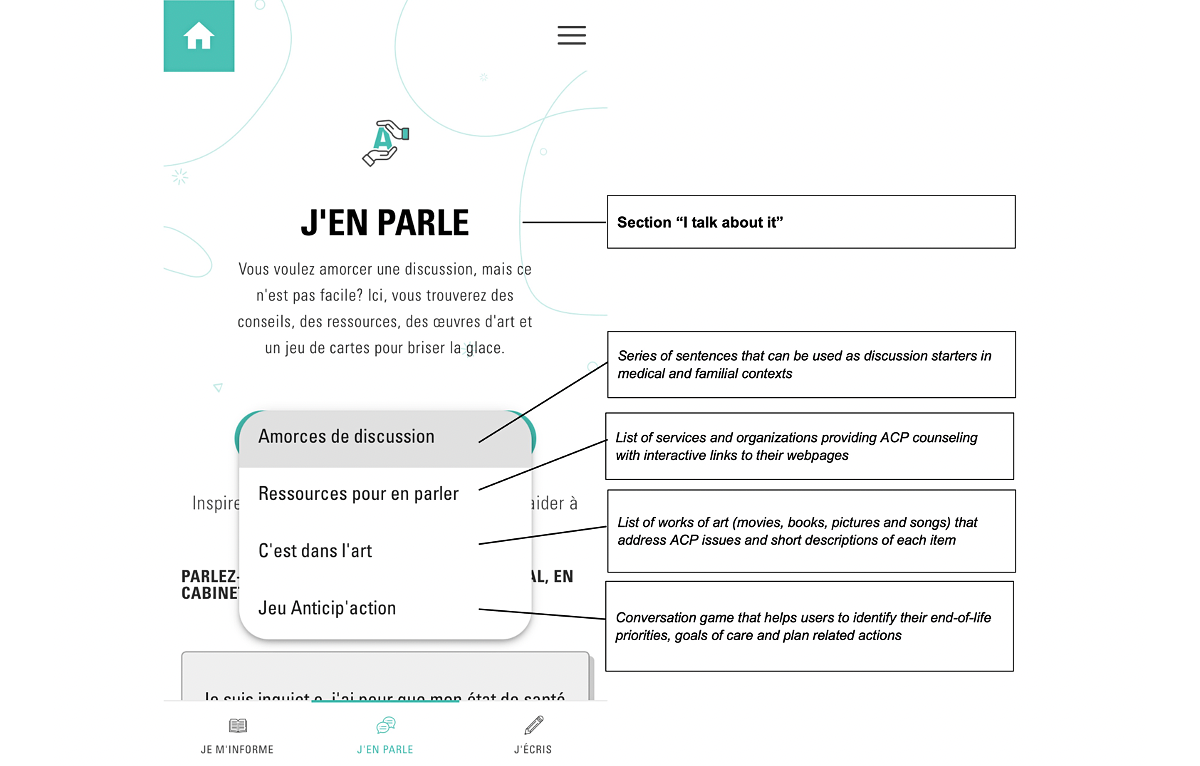
Structure of the section I talk about it with the different pages accessible through a drop-down menu. ACP: advance care planning.

**Figure 3:**
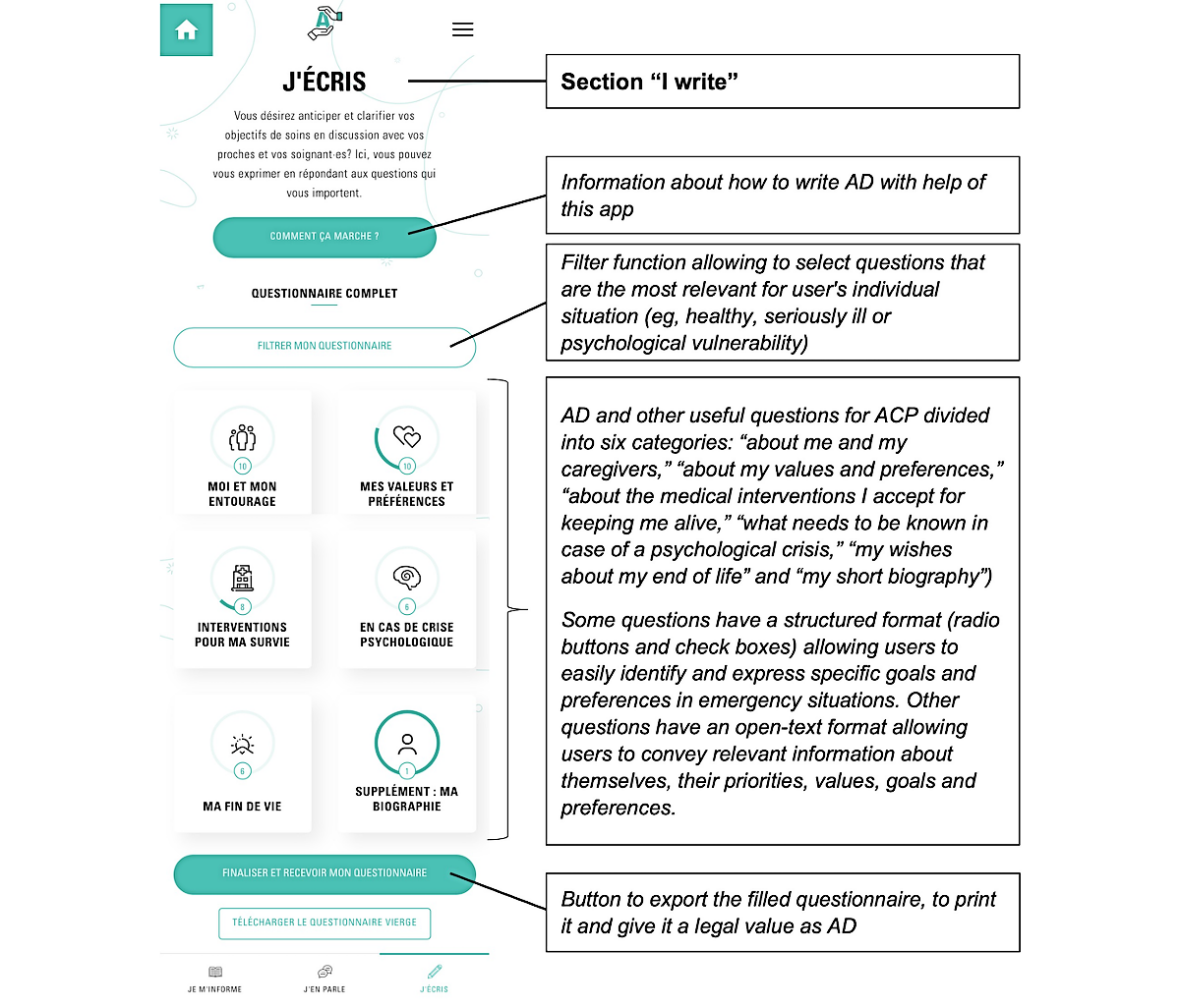
Structure of the section I write. ACP: advance care planning; AD: advance directives.

The app, developed in Angular 11 [26], is structured in 3 main sections (Figures 1-3)—*Je m’informe* (I get informed), *J’en parle* (I talk about it), and *J’écris* (I write)—accessible by clicking on a menu at the bottom of the screen. Each section contains several pages accessible via a drop-down menu.

### Study Objectives

The primary objective of this study is to measure whether *Accordons-nous* is easy to use and understand by common users. The secondary objectives are to identify the usability problems encountered by common users and to collect their critical opinions on the relevance of the tool—do they think that the app helps to engage in ACP procedures, and would they recommend the app to its target populations?

## Methods

### Participants

Nielsen and Landauer [27] reported that 98% of usability problems could be detected through feedback from 10 users. On the basis of this, we aimed to obtain at least 10 complete responses. We asked the *Patients as Partners Project* at the Geneva University Hospitals (Hopitaux Universitaires de Genève [HUG]) to recruit patients fulfilling our criteria. Participation was free and voluntary. The patients received and signed informed consent forms before the study.

We used the following inclusion criteria:

1. Fluency in written and oral French (the app is available only in French)
2. Comfortable with using a mobile phone or tablet
3. Demonstrates a minimal understanding of the concept of AD (to avoid a heavy cognitive load; ordinary users will have more time than our test participants to explore the app and be acquainted with the topic)
4. Did not participate in the development of *Accordons-nous* (to obtain unbiased responses)

Being incapable of understanding the meaning of the questions and tasks required for the study after 3 iterations of the task instructions was the only exclusion criterion.

### Ethical Considerations

We submitted our research protocol to the Geneva Commission Cantonale d’Ethique de la Recherche (Req-ID 2020-01397), who decided that the project does not fall within the scope of the Human Research Ordinance and therefore alleviated the need for ethical approval (decision date 12.1.2020). This study was conducted in accordance with the Declaration of Helsinki. Willing participants were informed of the study aims and procedures and signed an informed consent form. During the data collection and analysis phase, only three authors (CS, FE, and CC) had access to the recorded interviews. The recordings were destroyed after analysis. No identifying information was kept in the remaining work files.

### Study Intervention

We conducted a usability test [28] using a think-aloud method [29] comprising 7 tasks. Moreover, we collected quantitative descriptive data using a questionnaire to obtain information about participants’ endorsement of the app and their evaluation of the app’s usability and relevance for ACP.

We conducted the usability test sessions on the web using the Zoom video conferencing platform. Ahead of the test day, participants received the information and consent sheet and the instructions on how to log on to Zoom by email. Once connected, the experimenter (CS) recalled the procedure as described in the information and consent sheet, answered the participants’ questions, and asked for verbal (recorded on Zoom) or written (to be sent after the test) consent. The experimenter also expressed her commitment to respect the study protocol. Verbal consent was recorded separately to protect participants’ anonymity during the analysis stage.

Next, the experimenter instructed participants to install the test version of the *Concerto HUG* app that contains the module *Accordons-nous* on their personal smartphone or tablet. The experimenter then instructed the participants to connect their phone or tablet to the same Zoom conversation and share their phone or tablet screen. In this way, while discussing with the experimenter in front of their computer, participants could simultaneously navigate the app via their smartphone or tablet, and the experimenter could see how participants did so (owing to the screen sharing function). Precautions were taken beforehand to avoid personal notifications appearing during the screen sharing.

The experimenter then started the actual test. To ensure that participants fulfilled the inclusion criteria, she began by asking some demographic and technical questions (age, gender, and type of phone or tablet used), as well as participants’ understanding of the topic of AD (Multimedia Appendix 1; general questions).

Next, to ensure that participants did not need to express their personal choices and preferences regarding ACP, the experimenter asked the participants to put themselves in the following fictional scenario:

You are a 75-year-old man/woman; you have always been in good health, but you recently started experiencing a heart problem that required hospitalization. Fortunately, you recovered fully, but this experience made you think a lot. Now you are asking yourself, “What if another major health accident occurs, during which I lose my capacity to make decisions? How would health professionals know about what matters to me?” You know that it is possible to express your preferences in a document called “advance directives,” but you do not know how to do this. You share your concerns with a friend, who tells you about the application Accordons-nous and you decide to use it to write your directives. You have just downloaded the application and explore it for the first time.

Participants were then successively given 7 tasks to complete in the *Accordons-nous* module (Table 1). The tasks were designed to test users’ ease while completing the main tasks that a common user of the app is meant to handle and identify the strengths and weaknesses of the app.

While completing the tasks, participants were asked to express all their thoughts verbally (*think-aloud*), as if they were speaking to themselves. If participants forgot to share their reasoning aloud, the examiner reminded them to share their thoughts with her after 15 seconds. Otherwise, the examiner would not intervene. If the task was too complex and the participant felt lost for more than a minute or decided to give up, the examiner offered assistance by providing hints to help the participant move forward in solving the task. When required, the experimenter could provide four types of help: (1) information (eg, explanation of how the participant arrived where they are or why the module includes certain content), (2) motivating question (eg, “Right, and where would you go now?”), (3) answer (if the participant asked a question related to the exploration), and (4) guidance (eg, if the participant lingered too long on an irrelevant page, tell him or her what the next step would help find the answer).

At the end of each task, we asked participants, “Does the path to fulfil the task seems logical to you?” and “Do you have any suggestions for improvement?” to receive their qualitative feedback. Once the 7 tasks were completed, participants could easily browse the app at their ease if they wished to discover other functions or pages. To conclude, the experimenter asked a series of sociodemographic questions and questions that included items from two validated scales (System Usability Scale [SUS] [30] and Mobile App Rating Scale [MARS] [31]; Multimedia Appendix 1).

**Table 1:** List and description of the tasks used during the think-aloud test procedure.

Task name	Expected achievement	Exact wording of the question	Task considered as completed when
Definition	The participant discovers how to find definitions in the app.	“While researching advance directives, you heard the term ‘surrogate’ and want to know what it means exactly. Where would you look in the application to clarify its meaning?”	On the main page of the app, the participant clicks on the term *surrogates* (highlighted in green because it is a technical term) to pop up the definition; the participant finds the term on the *definitions* page of the app.
Legal obligations	The participant understands the logic of the green drop-down menu that allows accessing different pages within a section and finding specific legal information.	“You want to know what legal obligation doctors have toward patients who have written advance directives. Where would you go, in this application, to find this information?”	The participant finds the heading “Are doctors required to follow advance directives?” that is included in the page “What does the law say?” situated in the “I get information” section.
Conversation starters	The participant understands that the app is composed of 3 sections accessible via the menu at the bottom of the screen and finds specific content.	“You want to find ways to start a conversation with your son about your advance directives. Where would you go in this application?”	The participant discovered (1) the page *It’s in the art* or (2) the page *discussion starters* and verbalized that this could be used as a good conversation starter.
Data confidentiality	The participant finds the *How it works* pop up window that contains key elements for the process of writing advance directives and information on data confidentiality.	“After browsing the application for some time, you feel that you have enough information and decide to start writing your advance directives. However, before you complete the questionnaire, you want to know who will have access to the answers you write in the application. Where would you go in the module to get this information?”	The participant clicks on the button in the *I write* section and finds the relevant information.
Filter function	The participant discovers the filter function.	“You find that there are too many questions in the questionnaire, and you want to answer in a more targeted way the questions that are important to you. You would like to answer only the questions that concern healthy patients. How would you do this?”	The participant opens the filter function.
Values and preferences	The patient understands that the questionnaire is divided into several subsections and can make sense of their content by reading their title.	“Your faith in God is something central to your life. Therefore, it is really important for you to give details of your religious beliefs. Where would you go in the application to provide details on this matter?”	The participant finds question 15 “A few words about my spirituality” situated in the subsection “My values and preferences” of the questionnaire.
Finalize the questionnaire	The participant understands how to export from the app his or her answers to the questionnaire.	“You have answered all questions that are important to you and now you want to send your form by email. How would you do this?”	The participant clicks on the “Finalize and receive my questionnaire” button situated at the bottom of the questionnaire in the *I write* section.

### Measures Used and Data Analysis

#### Content Readability

On the basis of a document containing the full content (in text format) of the app, we evaluated its readability, page by page, with the help of Scolarius [32]. Influence Communication, a Canadian media analysis organization, developed this test. The test score is calculated in the same way as the Flesch Reading Ease score (often used for evaluating English material). It calculates the length of words and paragraphs. It provides a score ranging between 50 and 250 to be interpreted as follows: a score between 50 and 89 corresponds to an elementary school level of education, between 90 and 119 indicates a high school level, between 120 and 149 indicates a college level, and between 150 and 189 indicates a university level.

#### Tasks Evaluation

##### Overview

All the video recordings were double coded. CS ensured the first coding of all videos. CC and FE shared the task of the second coding. The recording of one participant was evaluated by 3 authors (CC, CS, and FE) to confirm unanimously that he met an exclusion criterion. Double codes were compared systematically. Whenever coders diverged in their evaluation, we applied the rules listed in Table 2.

**Table 2:** Rules used to arbitrate diverging evaluations in double coding.

Object of comparison and type of divergence		Rule applied
**Task success**		
	1-point difference between evaluations	Select the lowest score (to avoid desirability bias)
	>1 point difference between evaluations	Double check the recording and discuss the evaluation (CS, FE, and CC) until an agreement is reached
**Number of clicks**		
	≥1 point differences between evaluations	CS watches the video again to find the correct number
**Time spent on task**		
	Difference between the 2 evaluations <10%	Use the average time between the 2 evaluators
	Difference between the 2 evaluations >10%	CS watches the video again and decides
**Type of errors and problems**		
	Evaluators did not record the same errors and problems encountered by participants	Discuss the evaluation (CS, FE, and CC) until an agreement is reached
**Categorization**		
	Different categorizations for 1 error or problem	Discuss the evaluation (CS, FE, and CC) until an agreement is reached
**Severity rate**		
	Differences between evaluations of the severity rate of an error or problem	Discuss the evaluation (CS, FE, and CC) until an agreement is reached

##### Task Success

We calculated the number of participants who succeeded or failed to complete the tasks, with or without input from the experimenter. For this, we used the following scoring logic: 0=participant failed because the experimenter eventually gave the answer, 1=succeeded but did not use the shortest path and received some help from the experimenter, 2=succeeded without help but did not use the shortest path, and 3=succeeded without help and easily found the shortest path.

##### Clicks to Complete the Task

For each task, we reported the number of clicks needed to complete the task. Note that some tasks required more clicks than others; participants did not always start from the same page to complete the task; and for tasks 1 and 3, two answer paths were possible.

##### Time Spent on Task

For each task, we calculated the number of seconds required by the participants to complete it. Counting started when the experimenter finished formulating the question for the first time and ended when the participant successfully completed the required task (detailed in Table 1, right column). Whenever participants started to digress during task completion (eg, made critical comments on the design of the app or expressed a personal memory), this *digression time* was calculated and subtracted.

##### Errors and Problems Encountered

We recorded the errors and problems that the participants encountered while completing the 7 tasks. Each problem encountered was described in a Microsoft Excel file, categorized according to the heuristics of Bastien and Scapin [33] (information density, consistency, and significance of codes), and given a severity rate following the Nielsen Norman Group recommendations [27]: 0=disagreement that this is a usability problem at all, 1=cosmetic problem only—need not be fixed unless extra time is available, 2=minor usability problem: fixing this should be given low priority, 3=major usability problem: important to fix and should be given high priority, and 4=usability catastrophe—imperative to fix this before the product can be released.

##### Participants’ Feedback

Participants’ feedback after completing each task was coded as follows: 0=no, the path to complete the task makes no sense to me; 1=yes, it is more or less logical; and 2=yes, it is very logical. We took note of further comments made by the participants during the think-aloud tasks. We only reported comments that (1) were relevant to the objectives of the app and (2) contained proposals or ideas for improvements that could be realistically developed. We categorized comments as *useful for improving the app in the short term* or *useful for future developments*.

#### Questionnaire

##### Usability Assessment

To assess the app’s usability, we used the 10-item SUS questionnaire [30] suited for evaluating digital products. The SUS contains ten 5-point Likert score questions, allowing the calculation of a total score ranging between 0 and 100 (each question has a 10-point value). This indicates the effectiveness, efficiency, and overall ease of use of this app. A score of approximately 85 is considered excellent, approximately 72 is good, approximately 53 is acceptable, and approximately 38 is poor. Note that the first SUS question (“I think that I would like to use this system frequently”) is not relevant for the evaluation of *Accordons-nous*, as discussing end-of-life issues and writing AD is not an everyday activity. To address this issue, we added a slightly different first question to the 10 original SUS items (1bis), as follows: “I think that if I need to learn about advance care planning and want to write my advance directives, I would give priority to using this application.” This allowed the calculation of two SUS score: one with the standard scale; the other with question 1bis instead of question 1.

##### Relevance Assessment

To obtain participants’ evaluation of the impact of the app on the user’s awareness, knowledge, attitudes, intentions to change, and the likelihood of actual change in ACP behavior, we used the 6 *perceived impact* items (section F) of the validated MARS questionnaire [31]. The MARS questionnaire is particularly interesting as it was developed on the basis of the transtheoretical model of behavior change by Prochaska [13], which is commonly used in ACP literature to evaluate participants’ change in ACP engagement [34]. It allows calculating the overall mean 5-point Likert scores, to be interpreted as follows: 1=inadequate, 2=poor, 3=acceptable, 4=good, and 5=excellent.

##### Subjective Endorsement

To observe whether participants endorsed the app, we adapted the first 5-point Likert items of the MARS *subjective quality* (section E) questionnaire. We asked participants whether they would recommend this app to the following three target groups: patients, health professionals, and patients’ caring relatives.

## Results

### Participants

We obtained responses from 10 participants for all tasks and questions. During the study, we had to exclude 2 more participants: one because of technical problems (the sound recording did not work, making it impossible to analyze the results) and a second as he failed to understand 5 out of the 7 tasks that they were asked to perform, despite 3 iterations of the instructions.

Our data set included 60% (6/10) women and 40% (4/10) men of various ages (youngest: 31 years, oldest: 68 years; mean 50.8, SD 14.6 years). Of the 10 participants, 9 (90%) used devices running on iOS, and 1 (10%) used a device running on Android. All participants used these devices daily and showed minimal understanding of the notion of AD, thereby fulfilling our inclusion criteria.

### Content Readability

The overall Scolarius score of the app was 87.43/250, meaning that the app was readable for people with an elementary school level of education. However, some pages of the app require high school–level education. Table 3 provides the details of the score.

**Table 3:** Scolarius scores of the app Accordons-nous, per page.

Page of the app	Number of words	Score	Level of education
*L’essentiel* (key information)	254	100	High school
*Histoires de vie* (short life stories)	893	94	High school
*Que dit la loi?* (Swiss legal information)	876	89	Elementary school
*Définitions* (definitions)	1661	99	High school
*Amorces de discussion* (discussion starters)	312	60	Elementary school
*C’est dans l’art* (works of art)	3665	70	Elementary school
*I write* (filters and questionnaires)	3105	100	High school

### Tasks Evaluation

#### Task Success

**Figure 4:**
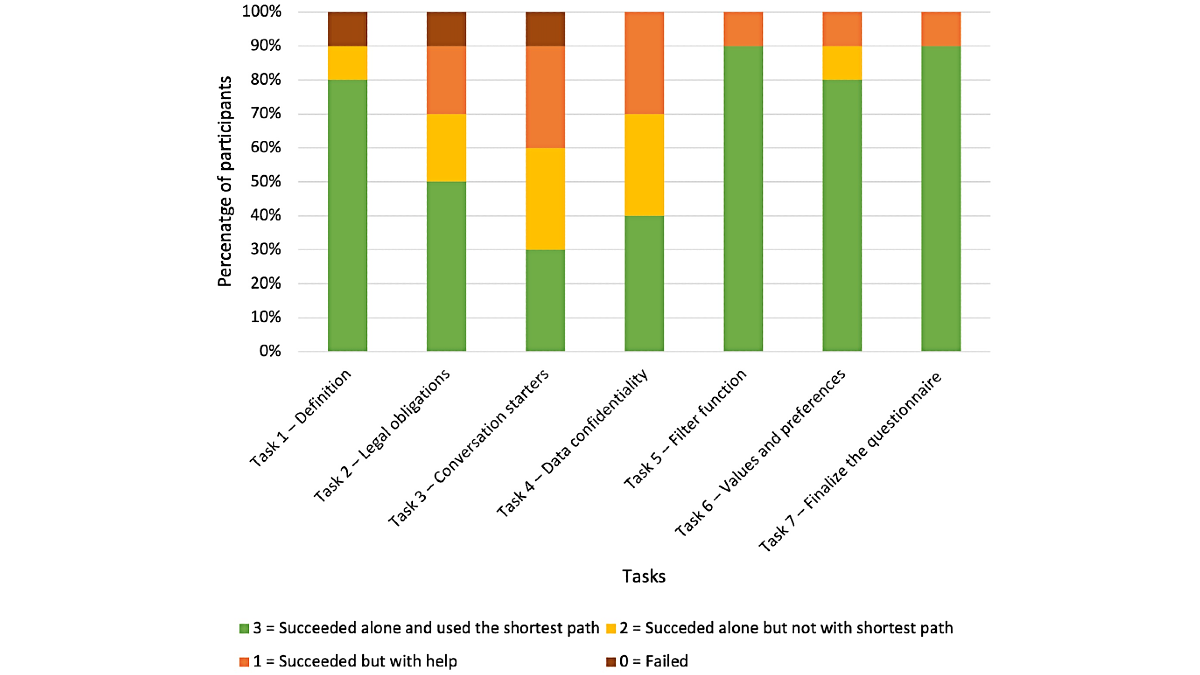
Proportion of participants who succeeded or failed to complete study tasks with or without input from the experimenter and with or without using the shortest route.

In 66% (46/70) of the cases, participants succeeded in completing a task without any help and easily found the shortest path (Figure 4). In 14% (10/70) of the cases, they succeeded without help; however, they did not take the shortest path. In 16% (11/70) of the cases, they succeeded without using the shortest route and needed some help from the experimenter. In 4% (3/70) of the cases, the experimenter eventually gave the answer. This 4% corresponds to 3 cases of failure, involving 30% (3/10) different participants. One failure on the task *definition* was obviously because this participant experienced a temporary state of panic at the beginning of the test and failed to grasp what the experimenter asked them to do (they struggled to find the right definition without looking at the app). In the case of the second failure, on the *legal obligations* task, the participant understood the logic of the drop-down menu within a section and therefore kept switching between the 3 main sections by using the bottom menu. The third failure was on the *conversation starters* task and seemed to be because of participants’ misunderstanding of the meaning of the three main sections: they reported having assumed that sections *I talk about it* and *I write* contained contact forms and therefore thought that this was not the place to find the conversation starters. Furthermore, once they discovered the section *I talk about it* (following guidance from the experimenter), they did not click on the green drop-down menu (a function that they had correctly discovered in task 1).

#### Clicks to Complete the Task

**Figure 5:**
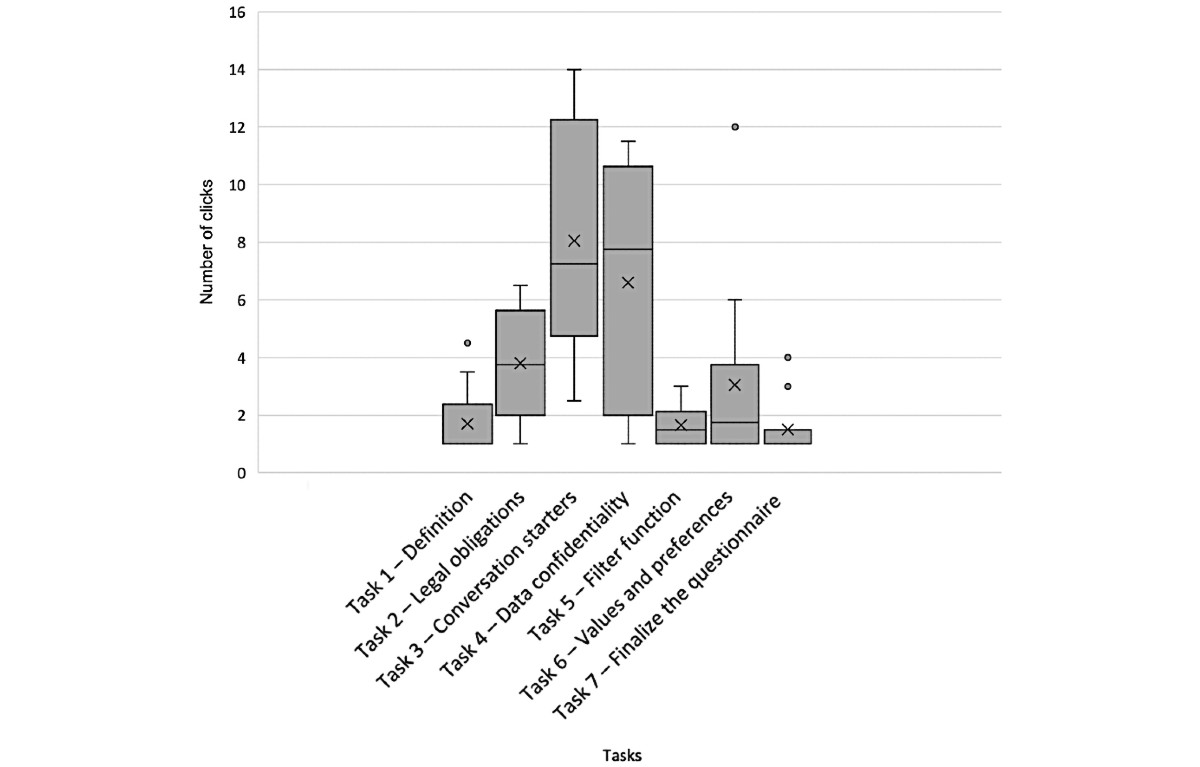
Distribution of the number of clicks needed for participants to complete each of the 7 tasks.

Participants’ actual number of clicks needed is illustrated in Figure 5. During the test, we noticed that the participants struggled to find a direct path toward data confidentiality information (task 4). We wondered whether this difficulty was affected by the order in which the questions were presented. To check this, in the last 2 interviews of the think-aloud procedure, we changed the order of the questions as follows: 1, 2, 3, 6, 4, 5, and 7. The change in order did not help to complete task 4 faster.

#### Time Spent on Task

**Figure 6:**
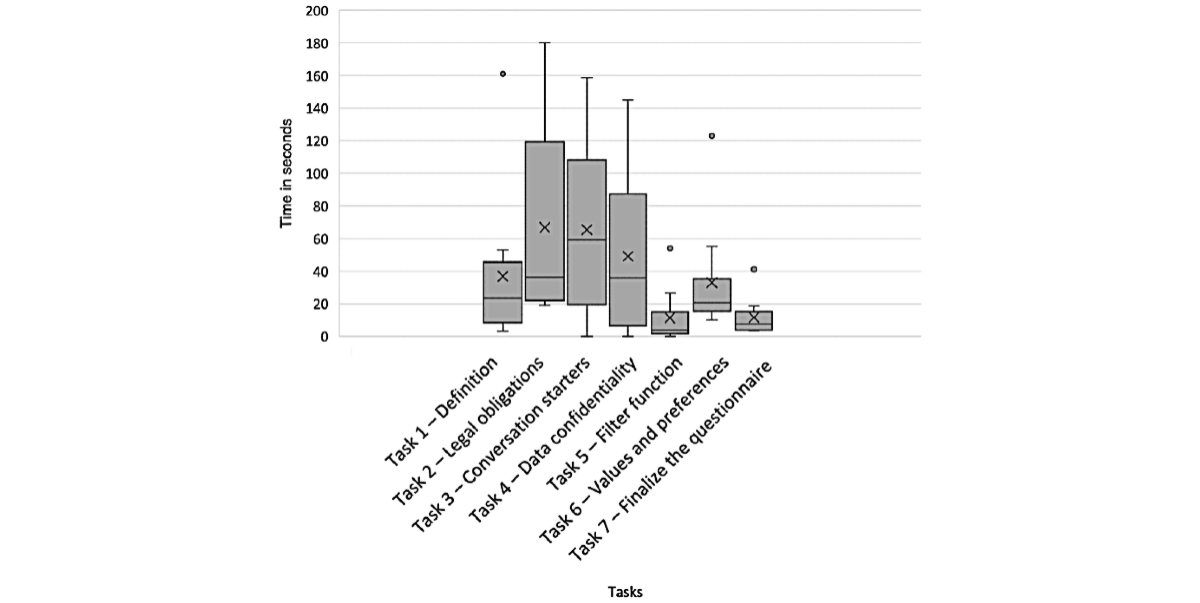
Distribution of time (in seconds) spent by participants completing each of the 7 tasks.

Participants’ actual time spent on the tasks is shown in Figure 6. Overall, these results indicate that participants encountered more difficulties in completing the tasks *legal obligations,* *conversation starters*, and *data confidentiality*. They encountered little difficulty in completing the last 3 tasks.

**Figure 7:**
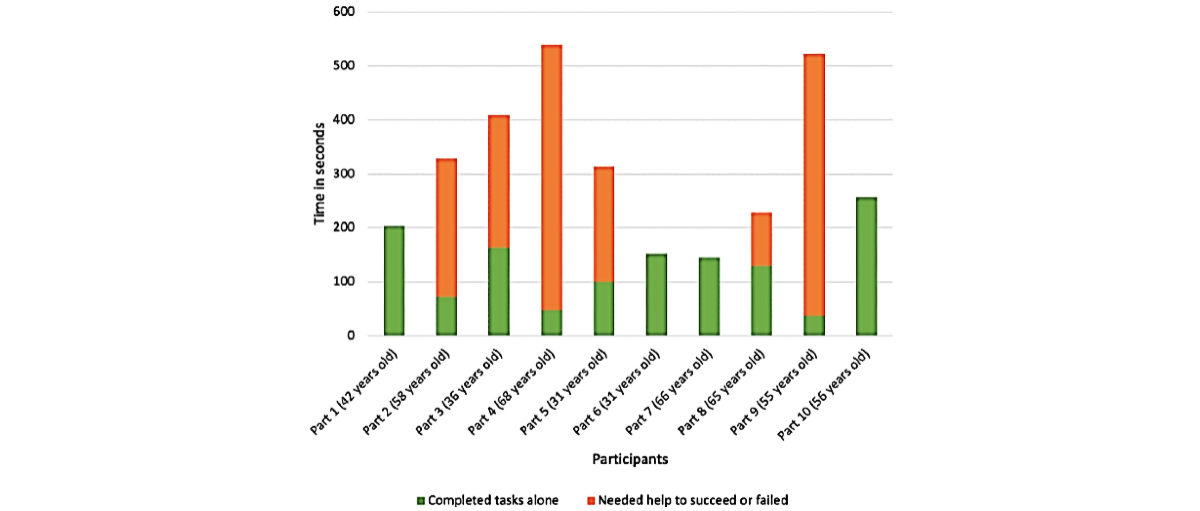
Total time (in seconds) spent by each participant on the 7 tasks of the think-aloud test. In green, time spent on tasks that have been completed successfully and without help (by using the shortest path or not). In red, time spent on tasks failed or completed with the help of the experimenter.

Figure 7 shows that there are important differences between participants: some participants navigated without difficulties, whereas others struggled on several tasks. Some participants needed much more time than others to complete their tasks; for instance, participant 10 used twice more time than participant 7 to successfully complete the tasks. Obviously, participants who needed help from the experimenter took much more time; for instance, participant 3 spent three-fourths of the whole test time on 1 task, for which they needed help.

The participants who succeeded in all tasks (4/10, 40%) were aged 31, 42, 56, and 66 years, indicating that the app is also understandable for older users.

#### Errors and Problems Encountered

In total, we recorded 44 problems encountered by the participants while completing the 7 tasks. Following the Bastien and Scapin [33] grouping method, we clustered them into 17 types of problems (Table 4). Of these problems, none were considered as a *usability catastrophe*, 50% (22/44) were considered as *major usability problems*, 48% (21/44) as *minor usability problems*, and the last 2% (1/44) as *not a usability problem at all*.

**Table 4:** List of problems encountered by participants during the 7 think-aloud tasks, classified in 3 severity categories (major usability problem, minor problem, and not a usability issue). Description, categorization, and frequency of occurrence of these problems during task completion.

Item and problem		Category (numbered according to Bastien and Scapin [33])	Description of the problem	Task in which it occurred (frequency of occurrence)
**Major usability problems (according to Nielsen Norman Group recommendations [27])**				
	1	1.2. Grouping or distinction of items	Users did not click on the green drop-down menu and therefore missed much of the content of the module.	Legal obligations (3); Conversation starters (1)
	2	7. Significance of codes	Users did not search for the information about the confidentiality of their AD^a^ in the right place: they felt that this information should have been located elsewhere in the app (for instance, in *I get information*, *What does the law say*, instead of *I write*, *How it works*, page).	Data confidentiality (5)
	3	1.3. Immediate feedback	Users expected feedback linked to the filter function in the *I write* section: the module did not provide feedback when a filter was selected. Therefore, users did not know whether their choice was taken into account.	Filter function (4)
	4	8. Compatibility	Participants tried to use functions they usually used in other apps; however, these were not active: one iOS user tried swiping left to return to the previous page, and an Android user used the back arrow to achieve the same goal; however, these functions were not active in *Concerto*.	Conversation starters (1); Data confidentiality (1)
	5	2.2 Information density	As the pages contained large sections of written content, some users took time to read the content of the pages, and this delayed their navigation or distracted them from the task. For instance, when they discovered the *It’s in the art* page, some users got *stuck* to discovering the different works of art.	Legal obligations (1); Conversation starters (2)
	6	1.4 Legibility	One user found that the bottom menu is too small and that the font does not offer sufficient contrast.	Conversation starters (1)
	7	7. Significance of codes	One user did not find the filter function explicit enough.	Filter function (1)
	8	8. Compatibility	One user was confused by the fact that on the first page, the title of the section *I get information* is larger than the title of the module.	Definition (1)
**Minor usability problems**				
	9	7. Significance of codes	Many users complained that they did not find the expected content by clicking on the hamburger menu at the top right of the screen: they thought that this would allow them to see the architecture of the module. However, this menu is not linked to the module *Accordons-nous* but to the host app, *Concerto*.	Definition (1); Legal obligations (2); Conversation starters (3)
	10	7. Significance of codes and 8. Compatibility	Many users clicked on the home menu at the top left of the screen, thinking that this would bring them back to the front page of the module *Accordons-nous*. However, this menu brought the user to the main menu of the host app, *Concerto*. Users were confused by this response and did not always understand where they had quit *Accordons-nous*.	Legal obligations (1); Conversation starters (2); Data confidentiality (1)
	11	8. Compatibility	Some users were looking for a main *home page* of the module; however, this page does not exist.	Definition (1); Data confidentiality (1); Values and preferences (1)
	12	7. Significance of codes and 8. Compatibility	Two users expected that the *I talk about it* and *I write* sections would work as a *contact us* page, which was not the case.	Conversation starters (2)
	13	1.4 Legibility	One user assessed the font size of the text as too small and the font contrast as insufficient.	Definition (1)
	14	1.2 Grouping or distinction of items	One user did not see the *How does it work?* button (section *I write*).	Data confidentiality (1)
	15	7. Significance of codes	One user did not see the *Filter function* button (section *I write*).	Filter function (1)
	16	8. Compatibility	One ordinary question in the AD form was confused with a function: a user thought that the question, “Where will you save your advance directives?” (multiple options are suggested as an incentive to store AD in several locations) would allow them to save the AD directly in different places.	Finalize questionnaire (1)
**Problem that we did not consider to be a usability issue**				
	17	8. Compatibility	One user complained that the app does not contain a search function.	Definition (1)

^a^AD: advance directives.

#### Participants’ Feedback

After completing a task, 64% (45/70) of the time, participants reported that the path to complete the task was *very logical*. In 31% (22/70) of the cases, they reported that it was more or less logical (the remaining 5% did not provide an answer). None of the participants found that the path to complete the task made no sense (Textbox 1).

**List of comments made by participants that are useful in the short and long term for improving the app.**

**Useful comments for short-term improvements**

**Figure 8:**
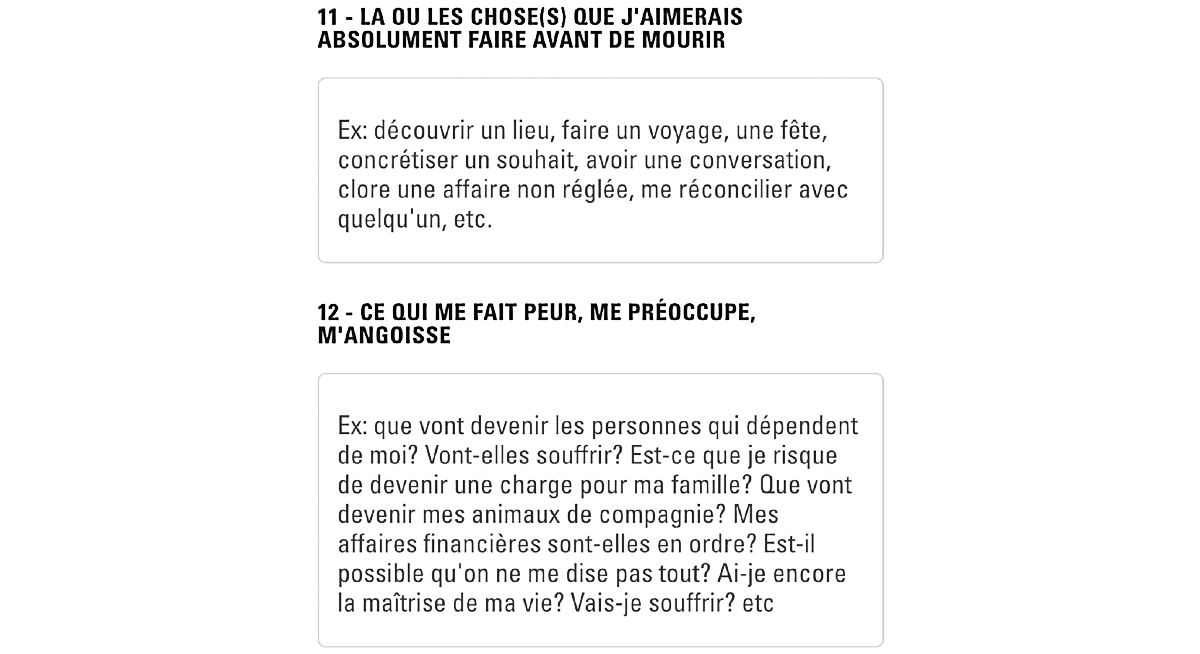
Two questions included in the advance directives (AD) form, with example responses in gray. The text disappears once the user clicks on the text box to add his or her own response.

1. Overall, 40% (4/10) of the oldest participants mentioned that “you have to look everywhere” to understand how to navigate.
2. Data privacy was a recurrent concern.
3. In total, 30% (3/10) of participants pointed out that the font was very small, which may be problematic for elderly users.
4. Many participants found that there might be too much to read in the app, especially given the fact that people tend to skip lengthy paragraphs.
5. Many participants said that they liked the example responses provided in the questionnaire (for illustration, see Figure 8). However, to avoid overloading the module with text, we included these texts in a lighter gray within the response box. The text disappears once the user clicks on the text box to add his or her own response. Some participants would have preferred these examples to remain visible (be placed above the box) to “not forget anything.”

**Useful comments for long-term improvements**

1. Some participants would have appreciated a search function.
2. With this app, a participant expected to be able to make calls or contact people around him or health professionals at the Hopitaux Universitaires de Genève.
3. A participant wished to have the function of electronic signature for signing the advance directives on the app.
4. A participant wished for more options for disabled patients, such as a dictation function for people who cannot write.

### Questionnaire

#### Usability Score

**Figure 9:**
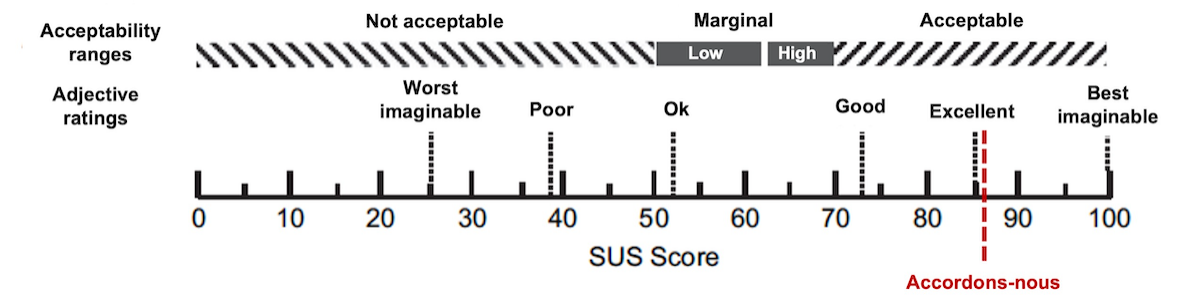
Score of Accordons-nous on the SUS. SUS: System Usability Scale [35].

As illustrated in Figure 9 [35], the app *Accordons-nous* scores very high on the SUS usability scale (85.25/100). When question 1 of the SUS was replaced with question 1bis, the total score increased to 90.5.

#### Relevance Score

Participants’ evaluation of the relevance of the app for supporting the process of ACP and AD is reported in Table 5. Most notably, 90% (9/10) to 100% (10/10) of the participants agree or strongly agree that *Accordons-nous* is likely to increase knowledge about the topic and the motivation to address the topic and induce related behavior changes. These responses lead to a high adherence rate, with an overall score of 4.27/5.

**Table 5:** Participants’ evaluation of the relevance of Accordons-nous for improving ACP^a^ and AD^b^ (N=10).

Measure	Question item	Responses, n (%)				
		Strongly agree	Agree	No opinion	Disagree	Strongly disagree
Awareness	This app is likely to increase awareness of the importance of (ACP and AD)	5 (50)	4 (40)	0 (0)	1 (10)	0 (0)
Knowledge	This app is likely to increase knowledge or understanding of (ACP and AD)	9 (90)	1 (10)	0 (0)	0 (0)	0 (0)
Attitude	This app is likely to change attitudes toward improving (ACP and AD)	3 (30)	4 (40)	3 (30)	0 (0)	0 (0)
Intention to change	This app is likely to increase intentions or motivation to address (ACP and AD)	4 (40)	5 (50)	1 (10)	0 (0)	0 (0)
Help seeking	Using this app is likely to encourage further help seeking for (ACP and AD)	1 (10)	5 (50)	3 (30)	1 (10)	0 (0)
Behavior change	Using this app is likely to increase behavior change (ACP and AD)	4 (40)	6 (60)	0 (0)	0 (0)	0 (0)

^a^ACP: advance care planning.

^b^AD: advance directives.

#### Subjective Endorsement

Overall, participants endorsed *Accordons-nous*. Of the 10 participants, 9 (90%) recommended the app (2 times 4/5 and 7 times 5/5 on the Likert scale) for patients. Only 10% (1/10) of participants *strongly disagreed* (1/5, lowest score) to recommend the app to patients, arguing that a smartphone app is not suitable for an older audience, whom they found as not being at ease with such technology. However, 90% (9/10) participants *strongly recommended* (5/5) the app to health professionals. Only 10% (1/10) disagreed (2/5), arguing that the tool was primarily designed for patients, and therefore, a professional would not find it relevant to their practice. Finally, all participants recommended (1 time 4/5 and 9 times 5/5) the app to family caregivers. The mean endorsement over the 3 questions was 4.7/5.

### Modifications Made Based on Results

The usability test revealed a series of issues that were addressed, as described in Table 6. *Accordons-nous* is an app in an app; that is, a module inserted in the larger host app *Concerto*. For this reason, some navigability issues could only be addressed by the *Concerto* team. Table 7 describes the issues that we reported to *Concerto* and how they have been addressed.

**Table 6:** List of changes made to the app in response to the major issues identified.

Item (Table 4)	Description of the issue	Changes made
1 and 6	Users did not click on the green drop-down menu and therefore missed much of the content of the module. One user found that the bottom menu is too small and that the font does not offer sufficient contrast.	To overcome navigation difficulties, we created a motion design video [36] (inserted on the first page of the app) containing navigation tutorial elements.
2	Users did not search for the information about the confidentiality of their advance directives in the right place: they felt that this information should have been located elsewhere in the app (for instance, in the *I get information*, *What does the law say*, instead of the *I write*, *How it works,* page).	We duplicated the information on “How is your privacy managed?” on the first page of the module.
3 and 7	Users expected feedback linked to the filter function in the *I write* section: the module did not provide feedback when a filter was selected; therefore, users did not know whether their choice was taken into account. One user did not find the filter function explicit enough.	First, we changed the color and location of the *How it works* button to make it more visible and added a video tutorial to explain how to write and store advance directives [23]. Second, we redesigned the filter page: we renamed and relocated the drop-down filter button to make it more explicit that it is a filter menu. Third, we added an automatic notification after the user has made a choice of filter.
5	As pages contained large sections of written content, some users took time to read the content of the pages, and this delayed their navigation or distracted them from the task. For instance, when they discovered the *It’s in the art* page, some users got *stuck* on discovering the different works of art.	First, we integrated 2 motion design videos containing illustrated summaries of the most important information. Second, we used the accordion visual presentation in sections containing large amounts of texts to help users to find the searched information without having to scroll through long texts. Third, in the *I talk about it* section, we preselected the entry page that contains less text to avoid a feeling of overload while navigating across sections.
8	One user was confused by the fact that on the first page, the title of the section *I get information* is larger than the title of the module.	We removed the title of the app on all pages.

**Table 7:** List of problems that were notified to the Concerto team and changes made.

Item (Table 4)	Reported problem	Change made
9	Several users clicked on the hamburger menu at the top right of the screen, thinking that this would allow them to see the architecture of *Accordons-nous*. However, this button is linked to the host app *Concerto* and provides only log-in information, which is confusing for the user.	The *Concerto* team removed the hamburger menu in *Accordons-nous* except when users are logged
10	Several users clicked on the home button at the top left of the screen, thinking that this would allow them to go back to the front page of *Accordons-nous*. However, this button brings the user to the front page of *Concerto*. This action involved quitting *Accordons-nous*, which also confused some of the users.	The *Concerto* team changed the design of the button: instead of a house, it is now the pictogram of the *Concerto* home page
4	Participants tried to use functions they usually used in other apps; however, these were not active: an iOS user tried swiping left to return to the previous page, and an Android user used the back arrow to achieve the same goal; however, these functions were not active in *Concerto*.	None to this date
13	The *Concerto* text font (imposed to all modules, including *Accordons-nous*) was judged as too small and not dark enough (most text is in a shade of gray).	None to this date

## Discussion

### Principal Findings

*Accordons-nous* is the first French-language mobile app for supporting the ACP process. In contrast to existing apps worldwide, which are limited in many respects [21], it includes materials and functions that facilitate each stage of the ACP process. It provides all the relevant information in an accessible language. It includes tools and prompts to facilitate contemplation and discussions about the goals of care and end-of-life issues. It provides guidance on writing or updating comprehensive and personalized AD in a simple process. In line with professional recommendations [22], it can be used as an icebreaker for starting discussions within families and as a follow-up to direct conversations with health professionals.

In our usability test, participants succeeded in the 7 think-aloud tasks in 80% (56/70) of the cases without help from the experimenter. However, in 16% (11/70) of the cases, help was needed, indicating that the app needed some improvement. An analysis of the navigational errors and difficulties encountered revealed no usability catastrophe but a series of major usability problems. These can be addressed with minor modifications (Table 6).

Our graphs indicate that participants performed better at completing the tasks over the course of the test, although the latter tasks were not necessarily easier to complete. We think that participants gradually became familiar and at ease with the logic of the app, which indicates that a few minutes of use are enough to grasp our tool. The easy-to-use character of the tool is particularly important [15] as ACP is mainly relevant for older patients [3] who are less used to digital devices. Our oldest participants (aged 66 and 68 years) fulfilled the think-aloud tasks well, indicating that age was not necessarily a limiting factor for using our tool. This is in line with previous results indicating that older patients are likely to use a platform for ADs with adequate design and support [15]. Moreover, as we designed *Accordons-nous* as a tool to help engage in discussions with family and health care providers, even patients who are not accustomed to digital devices may obtain support from surrounding care providers.

The app *Accordons-nous* obtained high scores on readability (overall score of 87.4 on the Scolarius test, corresponding to elementary school level). This is one of the best scores compared with other similar apps internationally [21]. However, the language difficulty varies across different pages of the app. Owing to the inherent complexity of the ACP topic, this difficulty can only be partially addressed; we ensured that all technical terms in the app were clickable to pop up the definition and added 2 introductory motion design videos summarizing the main information in simple terms and with illustrations.

Overall, our users provided very positive feedback on *Accordons-nous.* They assessed the app as likely to raise awareness of the importance of ACP and AD, increase knowledge of such topics, change attitudes and behaviors intention toward improving their own ACP, and encourage seeking further help to fulfill one’s ACP. Moreover, participants expressed a clear willingness to recommend the tool to relevant stakeholders, including patients, health professionals, and patients’ caring relatives. These are exciting results indicating that *Accordons-nous* is relevant and contains easily understandable content.

However, among the few critical views expressed, it is worth noting that 10% (1/10) of patients disagreed that *Accordons-nous* may encourage further help seeking in engaging in ACP, and 30% (3/10) had no opinion on that question. This result may indicate that patients expect the tool to offer the possibility of sending direct calls for help (eg, sending a message to a task force of professional ACP facilitators). Indeed, during the test, 20% (2/10) of participants erroneously thought at first glance that the section *I talk about it* would provide such direct support service. For practical and organizational reasons, we were not able to provide direct access to private counseling with *Accordons-nous*, which is a limitation of the app.

Our analysis of the main problems encountered by participants while completing the 7 think-aloud tasks indicates that some participants struggled with cognitive load: they failed a task or needed help to complete it as their attention was caught by the high density of information contained in some pages of the app. For instance, some participants got distracted by the long list of works of art that they discovered on the page *works of art*.

Other difficulties were because some participants did not spontaneously grasp the logic of navigating the app. In particular, some participants did not immediately see that the app contained 3 sections accessible via the navigation bar at the bottom of the screen (this was needed to complete the task *conversation starters*), whereas other participants encountered difficulties with the logic of the drop-down menu that allows navigating different pages (this was needed to complete tasks *legal obligation* and *conversation starters*). These difficulties indicate that some improvements needed to be made to the tool, especially for users who are not familiar with smartphone apps.

To address the main navigation difficulties and cognitive load because of the high information content, we included 2 introductory motion design videos in the app. In the first video [36], we explained the aim of the app, why ACP is useful, how the app is structured (3 sections), and the main navigation functions (menu to navigate the 3 main sections and drop-down menu buttons). In the second video [23], we explained how confidentiality issues are managed and how to write and store ADs with or without the help of the app.

Regarding task *data confidentiality*, several participants struggled to find the information as they searched it in the *I get informed* section rather than in the *I write* section. For this task, half of the participants reported that the path to complete it was *more or less logical* (rather than *very logical*). This indicates that data about confidentiality should be duplicated in both sections to be more easily found. This is particularly important as previous studies indicate that users are concerned about privacy issues [37]. We adapted the content of the app accordingly (Table 6).

Our results must be interpreted while considering the limitations listed in Textbox 2. Some limitations are common to usability studies, whereas others are minor issues related to small deviations from the original study protocol.

**List of identified limitations to our study.**

**Choice of method**

- As our test includes a small number of participants, no statistical conclusion can be drawn from this study.

**Sampling method**

- Owing to our sampling method (a small convenience selection of participants from the *Patients as Partners Project* at Hopitaux Universitaires de Genève), a courtesy bias [38] may have inflated positive results. Therefore, it is important to pay particular attention to the critical feedback provided.
- It may be that participants were more trained in medical matters than ordinary users, and this may have affected our results. On the other hand, ordinary patients would have much more time to browse and discover the app than our participants.

**Test conditions**

- Some failures or difficulties that we recorded may be because of the lack of time and stress induced by the test.

**Deviation from the study protocol**

- While writing the original protocol, we did not specify the exclusion criterion “Incapable of understanding the meaning of the questions and tasks required for the study.” However, we decided to exclude one participant on this criterion as he obviously did not grasp most of the tasks that we asked him to complete.
- During the test, we changed the order of the questions from 1-2-3-4-5-6-7 to 1-2-3-6-4-5-7 (see explanation in section *Results*, subsection *Clicks to Complete the Task*) The change of order did not help to complete task 4 faster. This change of order may have influenced the number of clicks to complete the tasks.

### Conclusions

The tool we developed is a novel solution for promoting ACP and AD. *Accordons-nous* is the first French-language mobile app developed by an interdisciplinary team of professionals in collaboration with target users. It includes a variety of content for prompting discussions related to medical emergency situations and end-of-life issues. It provides support for writing and easily updating AD on a smartphone or tablet. Considering the complexity and sensitivity of the process of ACP and given that we expect most users to be older people, we put special emphasis on producing easy-to-understand information, discussion prompts, and simple navigation principles. The results of our usability test with patients were very satisfying and helped us make the necessary final adjustments to our tool before making it available to the public. Further usability and efficacy tests involving health care professionals would help define whether the tool is also suitable for this population.

## Appendix

Multimedia Appendix 1 The history of the development of Accordons-nous and the list of questions submitted to the participants.

## Abbreviations

ACP: advance care planning
AD: advance directives
HUG: Hopitaux Universitaires de Genève
MARS: Mobile App Rating Scale
SUS: System Usability Scale
