# Supplementary material for: A Mobile App for Advance Care Planning and Advance Directives (Accordons-nous): Development and Usability Study
Source: JMIR Hum Factors. 2022 Apr 20;9(2):e34626. doi: 10.2196/34626 (PMC9069299; doi:10.2196/34626)
Supplement: Multimedia Appendix 1 [file humanfactors_v9i2e34626_app1.docx]

**Multimedia Appendix 1.** The history of the development of Accordons-nous and the list of questions submitted to the participants.

# Supporting information

*Accordons-nous*, A Mobile Application for Advance Care Planning and Advance Directives: Development and Usability Test

## Making of *Accordons-nous*

Our interprofessional team, composed of ethicists, physicians, nurses, information technology professionals, and patients as partners, developed the content and structure of the application *Accordons-nous* between January 2019 and July 2021, by using mixed methods: Delphi procedure and user tests.

To begin, we searched for existing ACP applications on Apple iTunes and Google Play stores. No application was available in French. We contacted 10 ACP academic experts and met them in individual meetings. They gave us preliminary reading and content inputs. Next to this, we conducted a broad scoping review of existing guidelines, important position papers, and ACP websites and applications available at international level.

Based on the above, we developed a detailed roadmap, including the content of the application and a first visual prototype of its structure. The roadmap was sent to an ACP expert panel ahead of a 24-hour Hackathon^44^, during which we scheduled a 1-hour workshop where we invited experts to provide their critical feedback. Our ACP expert panel (15 people) consisted of professionals with a background in medicine, nursing, psychology, philosophy, information or communication technology, and law, as well as research experience in the fields of palliative care, intensive care, oncology, nephrology, geriatrics, home care delivery, psychiatry, and ethics. During the Hackathon (March 2019), with the support of two professional IT developers and three patients as partners^45^ (members of the *Patients as Partners Project* at the HUG), we developed a first prototype in the form of a responsive app website.

After the hackathon, we extended our expert panel to more than 30 professionals, organised in-person meetings, exchanged via e-mail and finalised the development of a first version of the responsive app pilot. During this process, we also tested the pilot prototype with young and elderly lay people (each team member tested the tool with several family and friends and reported their feedback). We then organised a formal test day (September 2019), involving five patients recruited thanks to the HUG *Patients as Partners Project*.They were not previously acquainted with our project. Participants received and signed an information consent form ahead of the test. Based on a think aloud method^32^, participants were given a fictional scenario, in which they had to play the role of a person interested in ACP. This scenario helped patients to concentrate on the task of evaluating the application rather than on their personal attitude regarding the end of life. They were seated in separate rooms and asked to browse and discover the responsive app website on a tablet during a maximum of 45 minutes, while expressing aloud all their thoughts. Behind each participant, a research team member took notes on all relevant information (navigation difficulties encountered, comments made during the exploration phase) with the instruction not to interact with the patient except if he or she remained silent for more than 15 seconds or repetitively asked for help. Thereafter, participants answered a series of questions about the ease of navigation, their understanding of the content, and their critical views on the application and its various functions. One team member (CS) compiled all data and comments. We discussed them in subsequent group meetings and made further revisions to the prototype.

We then sent a link to the web prototype to our ACP expert panel along with an online questionnaire using Google Forms (December 2019). Experts could provide their critical feedback and recommendations on the content of the application (information and definitions provided, advance directive form, and other content) and on its structure (logic of navigation, layout, etc.). Again, we tested the pilot prototype with about 10 young and elderly lay people.

Throughout this multiple-stage process, proposals for adaptations were summarised by one team member (CS) and discussed within our working group with the use of interactive online working files and in-person group meetings where we also invited some experts. In most of these discussions and meetings, we included two patients as partners. Besides, research team members showed the prototype to lay people and reported their feedback to the group. We gradually refined the web app-responsive prototype up to a stage that we considered as completed. Based on this feedback, we made further fine-grained modifications to the prototype and provided all the necessary material to the HUG IT department for developing the application (February 2020).

Meanwhile, we started the internal validation process at HUG. The full text content of the application was sent to the *Patient and family information group* (GIPP), which includes doctors, nurses, and health communication specialists. This group is in charge of controlling that the content provided to the patient is easily understandable and appropriate. By August 2020, we received important feedback on wording and some elements of content (in particular, the legal aspects of advance directives) and made the necessary modifications.

Due to technical constraints related to the insertion of our application as a ‘module’ into the HUG patient application called *Concerto*, some navigation procedures and elements of structure had to be adapted. We also collaborated with the HUG Digital communication service for developing elements of design as well as motion design videos to integrate in *Accordons-nous*. At the end of the development procedure (December 2020), we sent an anonymous survey questionnaire to our ACP expert group, and to several additional lay people in order to evaluate the application’s navigability (questions to check whether users found the most relevant pages, menus, and functions), content clarity (with particular attention to some element, which we were unsure about), and to collect any further critical feedback. Some of our experts provided personal written assessments instead of filling in the questionnaire. 85.6% of the 15 respondents to the questionnaire reported no difficulty in navigating the module. Overall, no substantial critical comment was made, indicating that our tool was ready for dissemination.

In December 2020, we conducted the final think aloud usability test described in detail in this article, involving 10 more trained members of the HUG *Patients as Partners Project* that were not previously acquainted with our project. The results of the test (see below) generated a series of minor improvements. We could then submit the final content of the application to the *Patient and family information group* (GIPP). Further minor changes were suggested and integrated, and the application was submitted for final validation to the HUG M-health Committee (responsible for assessing and validating the quality of apps distributed under the responsibility of HUG), the HUG Health Director and Care Director (validation obtained in July 2021).

The official launch of *Accordons-nous* took place in December 2021. It is now available as an ‘app in app’, that is, a module inserted in the HUG *Concerto* application. *Concerto* provides several different services and can be downloaded for free on Apple iTunes store and Android Play store.

## Questionnaire

| **General questions (before think aloud tasks)** | | |
| --- | --- | --- |
| Number | Question item | Response options |
| 1 | Gender | Female; Male; Other |
| 2 | Age | number |
| 3 | What type of smartphone do you currently own? | IOS; Android; Other |
| 4 | How often do you use your smartphone? | Every day; Several times a week; Several times a month; A few times a year; Never |
| 5 | What is your understanding of advance directives? | open text |
| **System Usability Scale questionnaire** | | |
| Number | Question item | Response options  5pt Likert scale (1 - strongly disagree; 5 - strongly agree) |
| 1 | I think that I would like to use this system frequently. |  |
| 1bis | I think that if I need to learn about advance care planning and want to write my advance directives, I will give priority to using this application (instead of using other solutions provided by the Swiss Medical Association (FMH) or Pro Senectute). |  |
| 2 | I found the system unnecessarily complex. |  |
| 3 | I thought the system was easy to use. |  |
| 4 | I think that I would need the support of a technical person to be able to use this system. |  |
| 5 | I found that the various functions in this system were well integrated. |  |
| 6 | I thought there was too much inconsistency in this system. |  |
| 7 | I would imagine that most people would learn to use this system very quickly. |  |
| 8 | I found the system very cumbersome to use. |  |
| 9 | I felt very confident using the system. |  |
| 10 | I needed to learn a lot of things before I could get going with this system. |  |
| **MARS ‘perceived impact’ questions (section F)** | | |
| Measure | Question item | Response options  5pt Likert scale (1 - strongly disagree; 5 - strongly agree) |
| Awareness | This app is likely to increase awareness of the importance of [advance care planning and advance directives] |  |
| Knowledge | This app is likely to increase knowledge/understanding of [advance care planning and advance directives] |  |
| Attitude | This app is likely to change attitudes toward improving [advance care planning and advance directives] |  |
| Intention to change | This app is likely to increase intentions/motivation to address [advance care planning and advance directives] |  |
| Help seeking | Using this app is likely to encourage further help seeking for [advance care planning and advance directives] (if it is required) |  |
| Behavior change | Using this app is likely increase [advance care planning and advance directives] |  |
| **Subjective endorsement questions** | | |
| Number | Question item | Response options  5pt Likert scale (1 - not at all; 5 - definitely) |
| 1 | Would you recommend this application to patients [to engage in an advance care planning process and writing advance directives]? |  |
| 2 | Would you recommend this application to health professionals [to use it as a means of discussion with their patients]? |  |
| 3 | Would you recommend this application to family caregivers [to break the ice and address issues of advance care planning issues]? |  |
